# Supplementary material for: Chromosomal integration of blaCTX-M genes in diverse Escherichia coli isolates recovered from river water in Japan
Source: Curr Res Microb Sci. 2022 Jun 6;3:100144. doi: 10.1016/j.crmicr.2022.100144 (PMC9325909; doi:10.1016/j.crmicr.2022.100144)
Supplement: Supplementary file 1 [file mmc1.docx]

*Supplementary Information for*

**Chromosomal integration of bla_CTX-M_ genes in diverse Escherichia coli isolates recovered from river water in Japan**

Ryota Gomi^a*^, Masaki Yamamoto^b^, Michio Tanaka^b^, Yasufumi Matsumura^b^

^a^Department of Environmental Engineering, Graduate School of Engineering, Kyoto University, Katsura, Nishikyo-ku, 615-8540, Kyoto, Japan

^b^Department of Clinical Laboratory Medicine, Graduate School of Medicine, Kyoto University, 54 Shogoin-kawahara-cho, Sakyo-ku, 606-8507, Kyoto, Japan

^*^Corresponding author

E-mail address: gomi.ryota.34v@kyoto-u.jp; Tel: +81-75-383-3354; Fax: +81-75-383-3358

**Supplementary Materials and methods**

**Antibiotic susceptibility testing.**

Antibiotic susceptibility was assessed by microdilution using the dry plate Eiken assay (Eiken, Tokyo, Japan) using the antimicrobial agents listed in **Table S1**. The dry plate was designed to include antibiotic concentrations corresponding to both EUCAST epidemiological cutoff (ECOFF) values and CLSI breakpoints, where possible. We considered not only CLSI breakpoints (susceptible vs non-susceptible) but also ECOFF values (wild type vs non-wild type). This is because ECOFF values are epidemiologically based and thus are suitable for interpreting antibiotic susceptibility of environmental bacteria in comparison to clinical breakpoints, which are based on parameters relevant to therapeutic success. The dry plate was inoculated with an automated inoculation machine and incubated at 35 °C for 18 hours.

ESBL production was confirmed by disk diffusion using cefotaxime and ceftazidime disks with and without clavulanate (Eiken). The plate was incubated at 35 °C for 18 hours. A ≥ 5 mm increase in a zone diameter for either antimicrobial agent tested in combination with clavulanate compared with the zone diameter of the agent without clavulanate was considered ESBL-positive.

**Supplementary Figures**

**Figure S1.** Suggested mechanism for generation of context 1 and context 2 in KTa008. First, a 4,132 bp TPU was inserted into *dacD,* generating TSD of TATAT. Next, a 7,761 bp TPU, containing the 4,132 bp TPU and an adjacent region, was transposed into another chromosomal region, generating TSD of TATAA. Red arrows indicate antibiotic resistance genes, gray arrows indicate mobile elements, and white arrows indicate other genes. hp: hypothetical protein.

**Figure S2**. Genetic contexts of chromosomal *bla*_CTX-M_ genes in RefSeq environmental *E. coli* genomes. (a) The genetic context of *bla*_CTX-M-15_ in BEC1-S17-ESBL-09 (assembly accession no. GCF_014169915.1). A Tn*10*-like transposon was inserted into *yehB* in the chromosome, generating 9-bp TSD of CGCCTGGCG. Tn*2* was inserted in IS*10R* of the Tn*10*-like transposon, generating 5-bp TSD of TTATT. A 2,971 bp TPU containing *bla*_CTX-M-15_ was inserted into *tnpA* of Tn*2*, generating 5-bp TSD of TCATA. (b) The genetic context of *bla*_CTX-M-15_ in CF142 (assembly accession no. GCF_010365385.1). A 13,552 bp TPU, which carries *bla*_CTX-M-15_ and other resistance genes, was inserted in *recQ* in the chromosome, creating 5-bp TSD of TAATA. (c) The genetic context of *bla*_CTX-M-15_ in NMBU_W05E18 (assembly accession no. GCF_013282275.1). A 2,971 bp TPU carrying *bla*_CTX-M-15_ was inserted in IS*30*, generating 5-bp TSD of TAAGA. The TPU was surrounded by multiple ISs. (d) The genetic context of *bla*_CTX-M-24_ in SB0258h1 (assembly accession no. GCF_017603565.1). A 3,974 bp TPU carrying *bla*_CTX-M-24_ was inserted in an intergenic region in the chromosome, generating 5-bp TSD of GATTA. The references for the four genomes are as follows: BEC1-S17-ESBL-09 (Sekizuka et al. *Research Square*. 2020. DOI:10.21203/rs.3.rs-52275/v1.), CF142 (Bleichenbacher et al. *Environmental Pollution*. 2020. DOI: 10.1016/j.envpol.2020.115081.), NMBU_W05E18 (Finton et al. *Frontiers in Microbiology*. 2020. DOI: 10.3389/fmicb.2020.01273.), and SB0258h1 (Gray et al. *Microbiology Resource Announcements*. 2021. DOI: 10.1128/MRA.00328-21.).

**Supplementary Tables**

See the Excel file for the following tables:

**Table S1**. Basic characteristics of 18 ESBLEC isolates.

**Table S2**. Basic characteristics of each replicon of ESBLEC genomes sequenced to closure in this study.

**Table S3**. Results of BLASTN searches of TPU and the surrounding regions against the nr/nt database.

**Table S4**. Basic characteristics of RefSeq environmental *E. coli* genomes with *bla*_CTX-M_.
